# Application of multiplex ligation-dependent probe amplification, and identification of a heterozygous Alu-associated deletion and a uniparental disomy of chromosome 1 in two patients with 3-hydroxy-3-methylglutaryl-CoA lyase deficiency

**DOI:** 10.3892/ijmm.2015.2184

**Published:** 2015-04-14

**Authors:** YUKA AOYAMA, TOSHIYUKI YAMAMOTO, NAOMI SAKAGUCHI, MIKA ISHIGE, TOJU TANAKA, TOMOKO ICHIHARA, KATSUAKI OHARA, HIROKO KOUZAN, YASUTOMI KINOSADA, TOSHIYUKI FUKAO

**Affiliations:** 1 Medical Information Sciences Division, United Graduate School of Drug Discovery and Medical Information Sciences, Gifu University, Gifu 501-1194, Japan; 2 Department of Biomedical Sciences, College of Life and Health Sciences, Chubu University, Kasugai, Aichi 487-8501, Japan; 3 Tokyo Women’s Medical University Institute for Integrated Medical Sciences (TIIMS), Tokyo 162-8666, Japan; 4 Department of Pediatrics, Graduate School of Medicine, Gifu University, Gifu 501-1194, Japan; 5 Department of Pediatrics, Nihon University School of Medicine, Sapporo, Hokkaido 063-0005, Japan; 6 Department of Pediatrics and Clinical Research, NHO Hokkaido Medical Center, Sapporo, Hokkaido 063-0005, Japan; 7 Department of Pediatrics, Takamatsu Red Cross Hospital, Takamatsu, Kagawa 760-0017, Japan

**Keywords:** multiplex ligation-dependent probe amplification, mitochondrial 3-hydroxy-3-methylglutaryl CoA lyase, Alu element, uniparental disomy, microarray

## Abstract

Mitochondrial 3-hydroxy-3-methylglutaryl-CoA lyase (HMGCL) deficiency is an autosomal recessive disorder affecting the leucine catabolic pathway and ketone body synthesis, and is clinically characterized by metabolic crises with hypoketotic hypoglycemia, metabolic acidosis and hyperammonemia. In the present study, we initially used PCR with genomic followed by direct sequencing to investigate the molecular genetic basis of HMGCL deficiency in two patients clinically diagnosed with the condition. Although we identified a mutation in each patient, the inheritance patterns of these mutations were not consistent with disease causation. Therefore, we investigated *HMGCL* using multiplex ligation-dependent probe amplification (MLPA) to determine the copy numbers of all exons. A heterozygous deletion that included exons 2–4 was identified in one of the patients. MLPA revealed that the other patient had two copies for all *HMGCL* exons. Paternal uniparental isodisomy of chromosome 1 was confirmed in this patient by microarray analysis. These findings indicate that MLPA is useful for the identification of genomic aberrations and mutations other than small-scale nucleotide alterations. To the best of our knowledge, this is the first study describing HMGCL deficiency caused by uniparental disomy.

## Introduction

Mitochondrial 3-hydroxy-3-methylglutaryl-CoA lyase (HMGCL; EC 4.1.3.4) deficiency is an autosomal recessive disorder that affects leucine catabolism and ketogenesis. The *HMGCL* gene, located on chromosome 1p36.1, contains nine exons and spans approximately 25 kb (1). In the majority of HMGCL-deficient patients, the first hypoglycemic crisis occurs before the age of one, while one-third of all cases may have neonatal onset. In acute episodes, laboratory data have shown patients with non- or hypoketotic hypoglycemia with high levels of free fatty acids and severe metabolic acidosis with liver dysfunction and hyperammonemia (2). In Japan, HMGCL deficiency is one of the inborn errors of metabolism screened for in newborns by tandem mass spectrometry. Six Japanese HMGCL-deficient patients, including those previously reported (3) were re-evaluated (2). Among them, three had neonatal onset. Follow-up data showed that two patients experienced hypoglycemic crises even after ten years of age. Developmental delay and epilepsy were noted in two and three patients, respectively (2).

We recently encountered two Japanese HMGCL-deficient patients, whose inheritance patterns of single nucleotide mutations were not consistent with transmission within their families. *HMGCL* has 23 Alu elements in introns. Recombination between Alu elements results in genomic deletions associated with a number of human genetic disorders (4). Hence, we hypothesized that these patients may have an intragenic deletion by non-equal homologous recombination between Alu elements (5–7). Large homozygous deletions can be suspected by the absence of the deleted exons detected by PCR amplification. However, the detection of heterozygous deletions is difficult using routine PCR amplification of genomic DNA and direct sequencing. Multiplex ligation-dependent probe amplification (MLPA) has been proven to be an efficient and reliable technique for the copy number analysis of each exon in a gene (5,8–10). In the present study, we applied MLPA for the analysis of copy numbers in exons of *HMGCL* and confirmed mutations in the two patients with HMGCL deficiency.

## Patients and methods

### Patients

Patient 1 was of the female gender, born to non-consanguineous parents, who presented with hypoglycemia at the age of 2 days. She also experienced hypoketotic hypoglycemic crises at the age of 6, 8 and 13 months. She was diagnosed as having HMGCL deficiency at the age of 13 months by urine organic acid analysis, which detected 3-hydroxymethylgluta-rate, 3-methylglutaconate and 3-hydroxy-3-methylglutarate. The patient is currenlty 13 years old. She has epilepsy and developmental delay.

Patient 2 was of the male gender, born to non-consanguineous parents, who presented with vomiting and unconsciousness at the age of 3 months. He was diagnosed as having HMGCL deficiency at the age of 3 months by urine organic acid analysis and blood acylcarnitine analysis. He has experienced ten or more hypoketotic hypoglycemic crises, the last of which was at the age of 4 years. He is currently 8 years old and has achieved normal development. A case report for this patient has been previously published in Japanese (11).

### Mutation analysis at the genomic DNA level

The present study was approved by the Ethics Committee of the Graduate School of Medicine, Gifu University, Gifu, Japan. Genomic DNA was purified from peripheral blood samples using Sepa Gene kits (Sanko Junyaku Co., Ltd., Tokyo, Japan). Mutation screening was performed at the genomic level by PCR and direct sequencing, using a set of primer pairs that amplify fragments, including exons and their intron boundaries. The primer sequences are presented in Table I.

### Establishment of MLPA for the analysis of HMGCL

The MLPA reaction is an efficient and reliable technique for the analysis of exon copy numbers. We designed a pair of MLPA probes for each *HMGCL* exon, using the human MLPA probe design program (H-MAPD), as previously described (12). The MLPA probe sets for the *HMGCL* exon are listed in Table II. MLPA reactions were performed according to the manufacturer’s instructions (MRC-Holland BV, Amsterdam, The Netherlands) using 100 ng of genomic DNA, the EK1 MLPA reagent kit and the P200-A1 Human DNA reference kit, which includes reference probes and MLPA control fragments (MRC-Holland BV). The PCR products were separated by capillary electrophoresis on an ABI 3130×l genetic analyzer (Applied Biosystems, Warrington, UK). GeneMapper v4.0 software (Applied Biosystems) was used to analyze the separated products and to retrieve peak intensities corresponding to each probe in the different samples. Integrated peak areas were exported to an Excel 2003 spreadsheet. Data generated from a combination of the *HMGCL* synthetic probe mix and the P200-A1 probe mix were intra-normalized by dividing the peak area of the amplification product of each probe by the total area of only the reference probes in P200-A1. Secondly, normalization was achieved by dividing this intra-normalized probe ratio in a sample by the average intra-normalized probe ratio of all reference samples.

### Deletion breakpoint characterization

The region surrounding the deletion from intron 1 to intron 4 in patient 1 was amplified using three primer pairs as follows: fragment A: sense primer (In1s1, 5′-ACGAACGGTGGTAAAGAGGCAACAG-3′) located at position g.6421–6445 in intron 1 and antisense primer (Ex5as, 5′-TTGGCTGACTGCGCTGCCTTCAGGA-3′) located at position g.16255–16231 in exon 5; fragment B: sense primer (In1s3, 5′-GTGATGATTCCAGGAGGTCAGA GGA-3′) located at position g.8701–8725 in intron 1 and antisense primer Ex5as; and fragment C: sense primer In1s3 and antisense primer (In4as1: 5′-GAGAGGCATAGGACAGATTCTCC-3′) located at position g.15110–15088 in intron 4 (GenBank accession no. NG_013061).

PCR was carried out for 40 cycles at 94°C for 1 min, 64°C for 2 min, 72°C for 2 min followed by a 5-min extension at 72°C using Takara r-Taq (Takara Shuzo Co., Ltd., Shiga, Japan) and a Takara PCR thermal cycler. After subcloning into the pGEM-T Easy vector (Promega, Madison, WI, USA), the fragments were sequenced.

### Comparative genomic hybridization (CGH) and single nucleotide polymorphism (SNP) microarray analysis

Whole genomic copy number analysis was performed for patient 2 using an Agilent SurePrint G3 Hmn CGH + SNP 180K Microarray kit (Agilent Technologies, Santa Clara, CA, USA), according to the manufacturer’s instructions. Genomic DNA extracted from peripheral blood was used as a template. Data were extracted using Feature Extraction version 9 (Agilent Technologies) and the results were visualized using Agilent Genomic Workbench version 6.5 (Agilent Technologies).

## Results

### Mutation analysis

**Figure 1:**
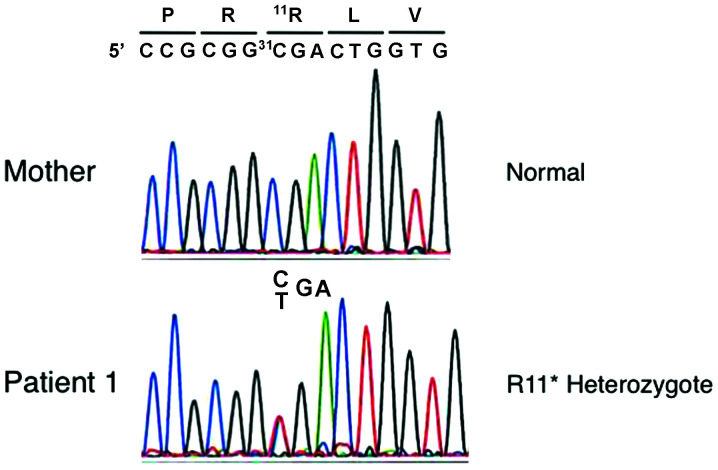
Sequence analysis of patient 1 and her mother. The c.31C>T (p. R11^*^) mutation site is shown.

**Figure 2:**
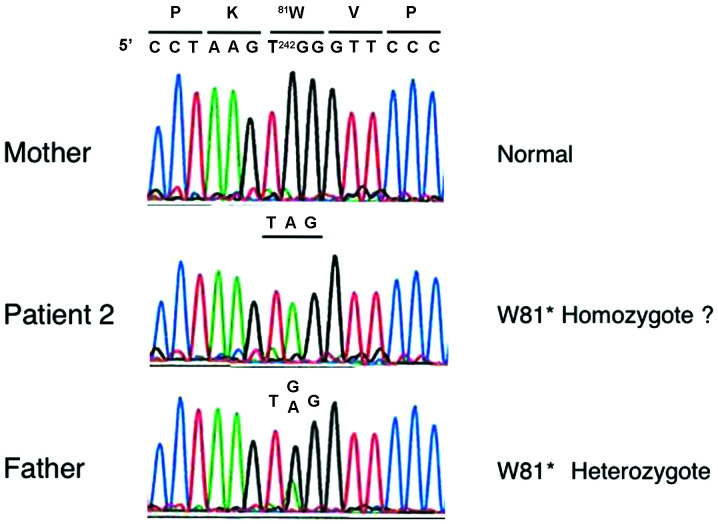
Sequence analysis of patient 2 and his parents. The c.242G>A (p.W81^*^) mutation site is shown.

Mutations were screened at the genomic level using PCR amplification followed by direct sequencing. Patient 1 had a heterozygous c. 31C>T (p.R11^*^) mutation in exon 1, and her mother did not have this mutation (Fig. 1). No mutation in the maternal allele was identified. The DNA of the father was not available. Patient 2 had a homozygous c.242G>A (p.W81^*^) mutation in exon 3. Genomic analyses of his patients revealed that the father was heterozygous for the p.W81^*^ mutation; however, the mother did not have the p.W81^*^ mutation (Fig. 2). Hence, we hypothesized the following: i) the maternal allele may have a large deletion not including exon 1 in patient 1; and ii) the maternal allele in patient 2 may have a deletion including exon 3.

### MLPA analysis of HMGCL

**Figure 3:**
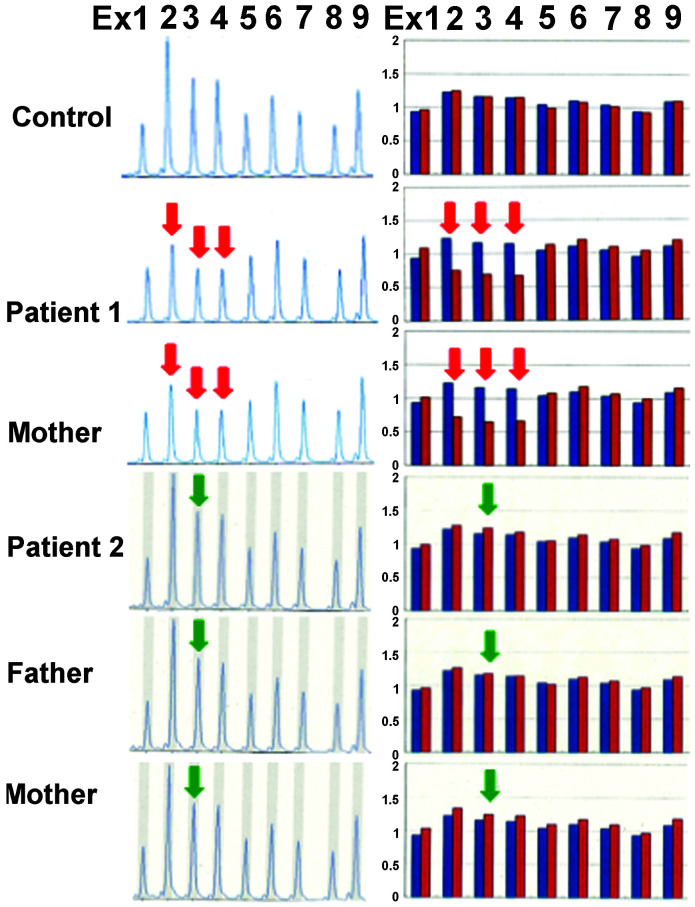
Multiplex ligation-dependent probe amplification (MLPA) of the mitochondrial 3-hydroxy-3-methylglutaryl-CoA lyase (*HMGCL*) gene. The MLPA profiles of each *HMGCL* exon are shown. The peaks derived from the P200-A1 reference probes followed by the peak of exon 9 are not shown in this figure. The histogram shows the calculated exonic dosage normalized as described in the Materials and methods. Blue and red columns indicate the dosage in the subject and in one control, respectively. Red arrows indicate that the bands had a significantly decreased dosage compared with the control, indicating that only one copy of exons 2–4 was present in patient 1 and her mother. Patient 2 and his parents had two copies of each exon including exon 3 (green arrows).

We successfully performed MLPA for *HMGCL*. Similar patterns of amplification were obtained in three controls, enabling copy number to be evaluated in the patient samples (Fig. 3). In patient 1 and his mother, only one copy of exons 2–4 was present, whereas two copies of all other exons were present, suggesting a large deletion including exons 2–4 in the maternal allele (Fig. 3). Patient 2 and his parents all had two copies of all *HMGCL* exons (Fig. 3). This means that both copies of exon 3 in patient 2 were from the father. We, therefore, suspected that patient 2 has a paternal uniparental disomy of the *HMGCL* region.

### Determination of breakpoints in patient 1

**Figure 4:**
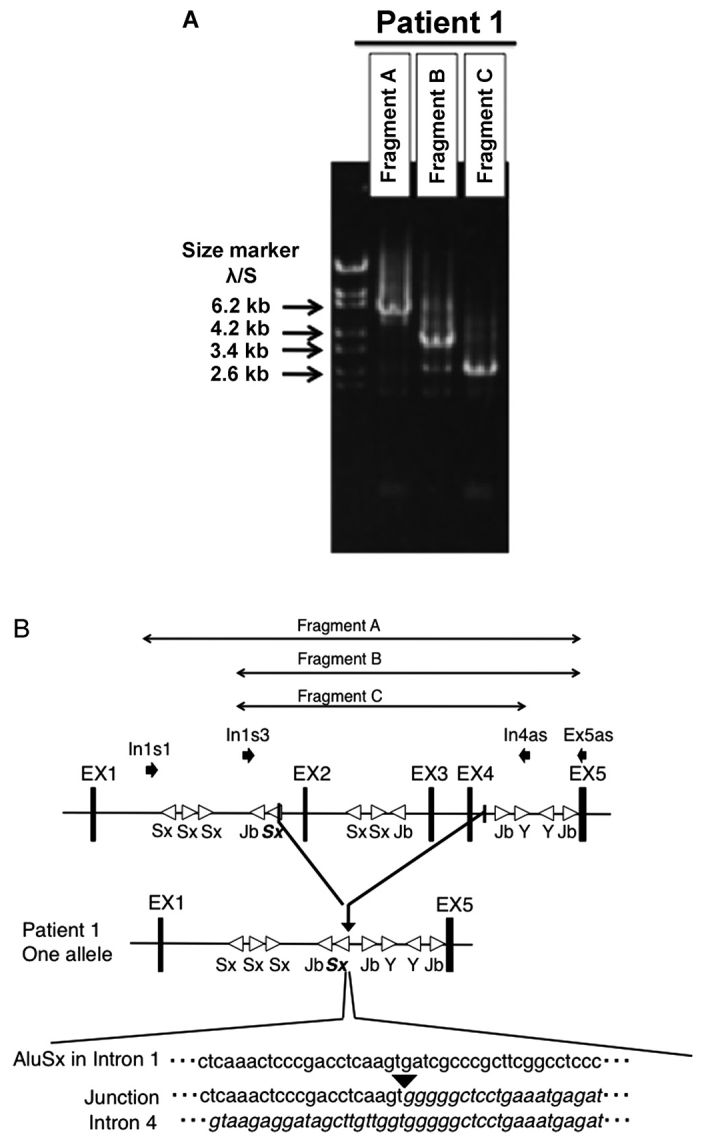
Characterization of a deletion that includes exons 3 and 4. (A) Long-range PCR. Fragments A, B and C were electrophoresed following amplification using genomic DNA and primer pairs of In1s1 and Ex5as, In1s3 and Ex5as, and In1s3 and In4as, respectively. The position of primers and their orientation are indicated in panel B. (B) Schematic representation of the rearranged mitochondrial 3-hydroxy-3-methylglutaryl-CoA lyase (*HMGCL*) gene in patient 1. Alu elements in introns 2–4 are indicated by arrowheads. Patient 1 had breakpoints within an Alu-Sx element in intron 1 and in a non-Alu sequence in intron 4, as indicated by the inverted triangle.

Long-range PCR amplification using DNA from patient 1 yielded fragments that included an approximate 4-kb deletion (Fig. 4A). A large deletion from g.9326 to g.13806 (4481 bp; NG_013061) was identified. We confirmed that one breakpoint was within an Alu element in intron 1 and the other was in a non-Alu element in intron 4 (Fig. 4B).

### CGH and SNP arrays in patient 2

**Figure 5:**
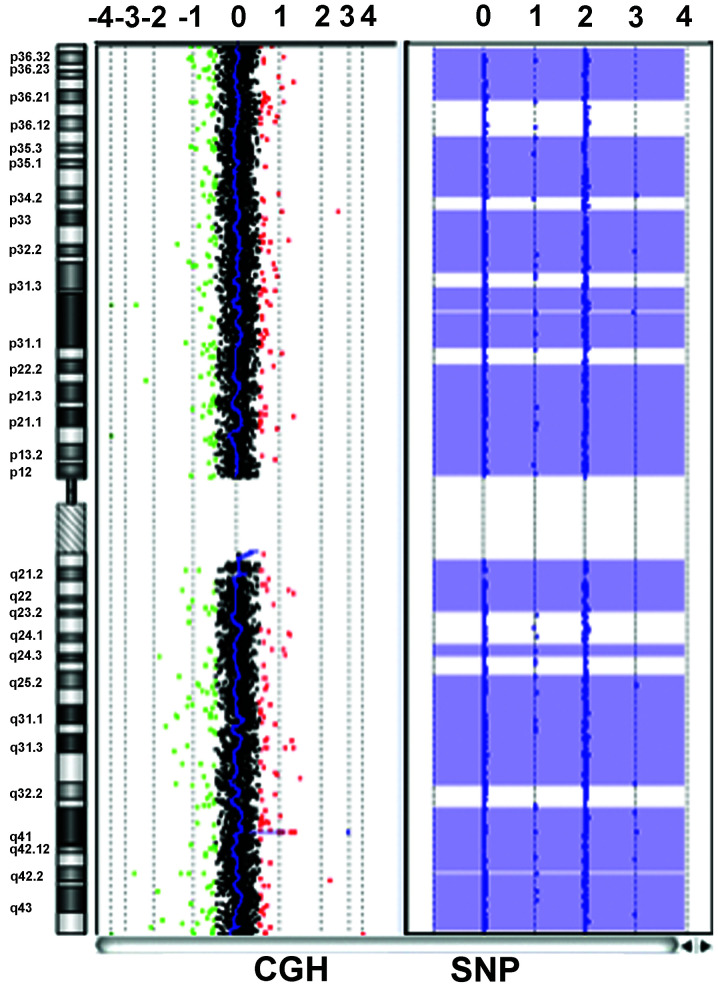
Results of chromosomal microarray testing using the comparative genomic hybridization and single nucleotide polymorphism (CGH + SNP) platform. The results of genomic copy number (left) and loss of heterozygosity (LOH) status (right) are shown for chromosome 1 of patient 2 according to the chromosome view of the Agilent Genomic Workbench. No genomic copy number aberration was detected in chromosome 1. Regions showing LOH status, judged by computer analysis, are depicted in purple; other non-LOH regions are considered to be false due to SNP artifacts. Patient 2 was, therefore, regarded to have LOH across the entire chromosome 1.

To confirm the copy number and SNP haplotype of the *HMGCL* region, microarray analysis using CGH + SNP array was performed for patient 2. In the CGH array, no copy number aberration was detected in the whole of chromosome 1 including the *HMGCL* region, which confirmed the homozygous status of the *HMGCL* mutation (Fig. 5).

Furthermore, a loss of heterozygosity (LOH) for almost all of chromosome 1, where *HMGCL* is located, was revealed. This indicated paternal uniparental disomy of chromosome 1. In conclusion, a homozygous mutation in *HMGCL* was determined to be caused by non-Mendelian inheritance due to paternal uniparental isodisomy of the whole chromosome 1, including the 1p36.1 region.

## Discussion

In the present study, we investigated the molecular genetic basis of two Japanese HMGCL-deficient patients using standard Sanger sequencing. Although we identified a mutation in each patient, inheritance patterns were not consistent in their families. Human *HMGCL* is an Alu element-rich gene, having 23 Alu elements within 23 kb. We first hypothesized that a heterozygous intragene deletion caused by non-equal homologous recombination between Alu elements was a possible cause of *HMGCL* mutation in these patients. Therefore, we used the MLPA method to detect the copy numbers of each exon in *HMGCL*.

Patient 1 had a heterozygous p.R11^*^ mutation from the father, but a mutation from the mother was not identified by conventional sequence analysis. MLPA revealed that patient 1 and her mother had only one copy of exons 2–4, which was consistent with our hypothesis. However, this large deletion that included exons 2–4 was not caused by non-equal Alu-mediated homologous recombination. One breakpoint was within an Alu element in intron 1 and the other was in a non-Alu element in intron 4. There were no homologous sequences around the breakpoint. The genesis of this deletion is unknown.

Patient 2 had an apparent homozygous p.W81^*^ mutation and his father was heterozygous for this mutation; however, his mother did not have this mutation. MLPA revealed that patient 2 had two copies of each *HMGCL* exon. Hence, our new hypothesis was that LOH of the *HMGCL* region may be the molecular basis for HMGCL deficiency in this patient. We successfully identified a paternal uniparental isodisomy of almost the entire chromosome 1 by microarray analysis using a CGH + SNP array. To the best of our knowledge, this is the first case of HMGCL deficiency shown to be caused by uniparental disomy.

There are two types of uniparental disomy, heterodisomy and isodisomy. In heterodisomy, a pair of non-identical chromosomes is inherited from one parent and in isodisomy a single chromosome from one parent is duplicated. Isodisomy is potentially dangerous as it may lead to the duplication of lethal recessive genes, while heterodisomy is essentially benign. Uniparental disomy in humans is mainly caused by meiotic non-disjunction events followed by i) gamete complementation; ii) trisomy rescue; iii) monosomy duplication; and iv) somatic crossing over (13). Our present results from *HMGCL* gene analysis, MLPA and CGH + SNP arrays indicate that patient 2 had a monosomic duplication of paternal chromosome 1. Partial and whole uniparental disomy of chromosome 1 has been reported in autism, fumarase deficiency, Stargardt disease, Pelizaeus- Merzbacher-like disease, leptin receptor deficiency, rhizomelic chondrodysplasia punctata type 2 and CD45-deficient severe combined immunodeficiency (14–20). This study demonstrates the advantage of using MLPA method for the analysis and identification of such heterozygous gene alterations. The identification of such mutations may facilitate mutation analysis in newly diagnosed patients.

## Figures and Tables

**Table I:** Amplification primer for *HMGCL* exons.

Exon	Foward primer	Sequence	Reverse primer	Sequence	Product size (bp)
1	HL1s	5′-GTGGAGCCAGCTTCGGAAGT-3′	HL1as	5′-GGGAGGGTCCAGGACTCCAACG-3′	324
2	HL2s	5′-ATGAATTCGGTCTCCCTGGGAATTG-3′	HL2as	5′-TAACTTGTGCAGAGGAATCACATC-3′	275
3	HL3s	5′-ATGAATTCTGCATTTTGAGGCTGTTT-3′	HL3as	5′-TTTGCTGCAACACAGTGCTATG-3′	325
4	HL4s	5′-ATGAATTCCTGCTCTTGGTGATGACT-3′	HL4as	5′-GATCACAGAGCAGTGAGTGGCA-3′	314
5	HL5s	5′-GAACCCAGGAGGTGGAGGTTGCA-3′	HL5a	5′-ATAAGCTTGAACGGTACAGAGGAAAGGA-3′	329
6	HL6s	5′-CTGGCACTGAATTGTACCAT-3′	HL6as	5′-GGGTGAATGAATGAAGTCAGGA-3′	336
7	HL7s	5′-AACTGAGTGCGTCATACCCAGA-3′	HL7as	5′-CAGAGCTGTACACTTCACATCTG-3′	473
8	HL8s	5′-ATGAATTCGGCAACAGACGATTGGG-3′	HL8as	5′-GAGCCACTGCGCCTGGCTAACC-3′	366
9	HL9s	5′-CCTGGTGTTGAGGGCATACC-3′	HL9as	5′-TGCCAGGAGAGACCTCTGTGTA-3′	300

**Table II:** MLPA probes for the *HMGCL* gene.

Exon	Product length (base)	Primer name	Length	Probe sequence
1	104	MLPA-HMGCLEX1L	50	GGGTTCCCTAAGGGTTGGA^5016^*TGGACTGCCGCGGGGGATTCTGGGCCAAGAT*
		MLPA-HMGCLEX1R	54	^5047^*GGCAGCAATGAGGAAGGCGCTTCCGCGGCGA*TCTAGATTGGATCTTGCTGGCAC
2	108	MLPA-HMGCLEX2L	52	GGGTTCCCTAAGGGTTGGA^9872^*CACCTCATCTATGGGCACTTTACCAAAGCGGGT*
		MLPA-HMGCLEX2R	56	^9905^*GAAAATTGTGGAAGTTGGTCCCCGAGATGGACT*TCTAGATTGGATCTTGCTGGCAC
3	112	MLPA-HMGCLEX3L	54	GGGTTCCCTAAGGGTTGGA^12925^*GAAGCAGGACTCTCTGTTATAGAAACCACCAGCTT*
		MLPA-HMGCLEX3R	58	^12960^*TGTGTCTCCTAAGTGGGTTCCCCAGGTGAGCCCTA*TCTAGATTGGATCTTGCTGGCAC
4	116	MLPA-HMGCLEX4L	56	GGGTTCCCTAAGGGTTGGA^13730^*TTCCTGGCATCAACTACCCAGTCCTGACCCCAAATTT*
		MLPA-HMGCLEX4R	60	^13767^*GAAAGGCTTCGAGGCAGCGGTAAGAGGATAGCTTGTT*TCTAGATTGGATCTTGCTGGCAC
5	120	MLPA-HMGCLEX5L	58	GGGTTCCCTAAGGGTTGGA^16193^*TTGTTCCATAGAGGAGAGTTTTCAGAGGTTTGACGCAAT*
		MLPA-HMGCLEX5R	62	^16232^*CCTGAAGGCAGCGCAGTCAGCCAATATTTCTGTGCGGGG*TCTAGATTGGATCTTGCTGGCAC
6	124	MLPA-HMGCLEX6L	60	GGGTTCCCTAAGGGTTGGA^19636^*TCATTCCTCCCCTGTCTTCCCACAGGTACGTCTCCTGTGCT*
		MLPA-HMGCLEX6R	64	^19677^*CTTGGCTGCCCTTATGAAGGGAAGATCTCCCCAGCTAAAGT*TCTAGATTGGATCTTGCTGGCAC
7	128	MLPA-HMGCLEX7L	62	GGGTTCCCTAAGGGTTGGA^22152^*TACTCAATGGGCTGCTACGAGATCTCCCTGGGGGACACCATTG*
		MLPA-HMGCLEX7R	66	^22195^*GTGTGGGCACCCCAGGGATCATGAAAGACATGCTATCTGCTGT*TCTAGATTGGATCTTGCTGGCAC
8	132	MLPA-HMGCLEX8L	64	GGGTTCCCTAAGGGTTGGA^25930^*TCTAGATGGGAGTGAGTGTCGTGGACTCTTCTGTGGCAGGACTTG*
		MLPA-HMGCLEX8R	68	^25975^*GAGGCTGTCCCTACGCACAGGGGGCATCAGGAAACTTGGCCACAG*TCTAGATTGGATCTTGCTGGCAC
9	136	MLPA-HMGCLEX9L	66	GGGTTCCCTAAGGGTTGGA^2797^*5CTCAGGCTACCTGTAAACTCTGAGCCCCTTGCCCACCTGAAGCCCTG*
		MLPA-HMGCLEX9R	70	^28022^*GGGATGATGTGGAAATAGGGGCACACACAGATGATTCATGGATGGGG*TCTAGATTGGATCTTGCTGGCAC

Left primer sequence (5′→3′), GGGTTCCCTAAGGGTTGGA; right primer sequence (5′→3′), TCTAGATTGGATCTTGCTGGCAC. Hybridization sequences are shown in italics. MPLA, multiplex ligation-dependent probe amplification.

## Abbreviations

HMGCL: 3-hydroxy-3-methylglutaryl-CoA lyase
MLPA: multiplex ligation-dependent probe amplification
CGH: comparative genomic hybridization
SNP: single nucleotide polymorphism
LOH: loss of heterozygosity
